# Die Störungen des Verdauungsapparates als Ursache und Folge anderer Erkrankungen

**Published:** 1899-09

**Authors:** 


**Die Störungen des Verdauungsapparates als Ursache und Folge anderer Erkrankungen.** Von Dr. HANS HERZ. Pp. xviii., 543. Berlin: S. Karger. 1898.

The plan of this book is original, and we know of no work in English that covers exactly the same ground. The author's idea is to consider the disorders of the alimentary tract and its accessory glands as they occur in the course of general diseases and of diseases of other organs. In this way a broad general view may be arrived at of the modes in which the digestive apparatus is affected in diseases which have no causal connection with it. At the same time the reverse of the picture is taken into view, and the digestive organs considered as the seat of origin of disease. Thus throughout the volume two points of view are consistently regarded: firstly, the alimentary tract considered as the primary seat of each disease or group of diseases; and secondly, the secondary disturbances of this tract which occur in the same diseases when they own another source of origin. We may take the acute infectious diseases as an example. The share of the digestive tract in the etiology of these diseases is first discussed, with an appendix on the occurrence of fever in disorders of the alimentary canal, and then the secondary disturbances of this tract which occur in the course of these fevers are systematically described and grouped under the heads of symptoms referable to the mouth, pharynx, stomach, intestines, liver, spleen, pancreas, peritoneum, and mesenteric glands. Again, in syphilis and tuberculosis the clinical and pathological history is given of the cases in which these infections obtain entrance to the system through the alimentary canal, and then of the secondary disturbances of the latter when an entrance of the poison is gained through some other channel.

The author points out that although much has been and is still to be gained by differentiation and distinction in the consideration of diseases, less has been done—and there is probably almost as much information to be gathered—by putting together like things. His book is to be regarded as an effort in this direction, a contribution to homogeneity in medicine, an example of how light may be thrown on obscure processes of disease in one system of organs by comparing their disorders as they occur in different diseases. Nor is it to be supposed, and the work under notice is a striking example to the contrary, that such a consideration of the disorders of one system by itself must necessarily lead to a narrow specialism, to a limited grasp or one-sided view of the problems of medicine. Rather the effect should be to widen and enlarge one's ideas, for in this particular instance of the digestive system, as one reads on one realises with increasing force how intimately this system is bound up with the rest, and how impossible it is to treat its disorders scientifically without a full appreciation of the mutual interdependence of organs upon each other, and of the fact that no one system can be disturbed without affecting to a greater or less degree every other; that, in clinical language, the patient and not the disease is to be treated.

Enough has been said to show that this book is written in a scientific spirit, and approaches its subject from an aspect that is novel and capable of throwing new light upon what may be already familiar to the reader. But in addition there must be very much that is actually fresh to most readers, and more that is new in the sense that it is now first collected in a readily accessible form. Obviously the labour of writing a book dealing with diseases of the digestive apparatus on such a plan must be very great, and the author displays in the prosecution of it immense erudition and research. When we consider the extensive literature of the primary digestive disorders by themselves alone, and then that of the secondary disorders as met with in all other diseases, we can appreciate the magnitude of the task. So far as we have been able to ascertain, the author has carried out his plan remarkably well, and his work is a storehouse of recorded facts and observations on the pathology and symptoms of every affection of the alimentary tract. It thus forms a valuable work of reference which contains a quantity of information that otherwise would have to be laboriously sought out in scattered papers. Not that we mean to imply that the book is a mere compilation; far from it,—it is written with critical insight, and bears the stamp of experience and sound judgment. There is one point on which we disagree with the author; in his preface, he states that he considers an index unnecessary, as he has given a very full table of contents. We think, however, that to bring out the full value of his book as a work of reference, it requires a copious index as well.

